# Epigenetic Aberration of *FMR1* Gene in Infertile Women with Diminished Ovarian Reserve

**DOI:** 10.22074/cellj.2018.4398

**Published:** 2018-01-01

**Authors:** Hossein Eslami, Ali Eslami, Raha Favaedi, Ummolbanin Asadpour, Shabnam Zari Moradi, Poopak Eftekhari-Yazdi, Tahereh Madani, Maryam Shahhoseini, Anahita Mohseni Meybodi

**Affiliations:** 1 Department of Biology, Faculty of Science, Science and Research Branch Islamic Azad University, Tehran, Iran; 2 Department of Genetics, Reproductive Biomedicine Research Center, Royan Institute for Reproductive Biomedicine, ACECR, Tehran, Iran; 3 Department of Embryology, Reproductive Biomedicine Research Center, Royan Institute for Reproductive Biomedicine, ACECR, Tehran, Iran; 4 Department of Endocrinology and Female Infertility, Reproductive Biomedicine Research Center, Royan Institute for Reproductive Biomedicine, ACECR, Tehran, Iran

**Keywords:** Epigenetic, *FMR1* Gene, Histone Modification, Ovarian Reserve Cell Journal(Yakhteh), Vol 20, No 1, Apr-Jun (Spring) 2018, Pages: 78-83

## Abstract

**Objective:**

The diminished ovarian reserve (DOR) is a condition characterized by a reduction in the number and/or quality of oocytes. This primary infertility disorder is usually accompanied with an increase in the follicle-stimulating hormone (FSH) levels and regular menses. Although there are many factors contributing to the DOR situation, it is likely that many of idiopathic cases have genetic/epigenetic bases. The association between the *FMR1* premutation (50-200 CGG repeats) and the premature ovarian failure (POF) suggests that epigenetic disorders of *FMR1* can act as a risk factor for the DOR as well. The aim of this study was to analyze the mRNA expression and epigenetic alteration (histone acetylation/methylation) of the *FMR1* gene in blood and granulosa cells of 20 infertile women.

**Materials and Methods:**

In this case-control study, we analyzed the mRNA expression and epigenetic altration of the *FMR1* gene in blood and granulosa cells of 20 infertile women. These women were referred to the Royan Institute, having been clinically diagnosed as DOR patients. Our control group consisted of 20 women with normal antral follicle numbers and serum FSH level. All these women had normal karyotype and no history of genetic disorders. The number of CGG triplet repeats in the exon 1 of the *FMR1* gene was analyzed in all samples.

**Results:**

Results clearly demonstrated significantly higher expression of the *FMR1* gene in blood and granulosa cells of the DOR patients with the *FMR1* premutation compared to the control group. In addition, epigenetic marks of histone 3 lysine 9 acetylation (H3K9ac) and di-metylation (H3K9me2) showed significantly higher incorporations in the regulatory regions of the *FMR1* gene, including the promoter and the exon 1, whereas tri-metylation (H3K9me3) mark showed no significant difference between two groups.

**Conclusion:**

Our data demonstrates, for the first time, the dynamicity of gene expression and histone modification pattern in regulation of *FMR1* gene, and implies the key role played by epigenetics in the development of the ovarian function.

## Introduction

One of the well-known causes of female infertility is the diminished ovarian reserve (DOR). DOR is characterized by a reduction in the number and/or quality of oocytes, low likelihood of establishing a pregnancy, increased miscarriage rates, and poor response to ovarian stimulation in *in vitro* fertilization (IVF) (1, 2). The prevalence of DOR has been estimated to be approximately 10% among young women (3, 4). Despite its prevalence, its etiology remains a mystery. Aging is the most common cause of diminished ovarian reserve. Other reasons for the diminished ovarian reserve include chemotherapy, radiation therapy, autoimmune diseases, and certain genetic conditions (5). 

The fragile X mental retardation 1 gene (*FMR1*) is located at Xq27.3 and is responsible for the fragile X syndrome, a form of X-linked mental retardation. This disorder is caused by the expansion of a polymorphic CGG trinucleotide repeat in the promoter of the *FMR1* gene, consisting of more than 200 repeats (full mutation), instead of the usual 6-54 CGG repeats (6, 7). This trinucleotide expansion induces methylation of cytosines within the CpG islands inside the repeat tract and in the flanking sequence, including the *FMR1* gene promoter, resulting in the epigenetic inactivation of the gene, which in turn switches off the production of the *FMR1* protein (FMRP) (8-10). Premutation alleles (55-200 CGG repeats) have been associated with premature ovarian failure (POF).

It has been reported that the rearrangements of the X chromosome are associated with the POF (11). Two main critical regions in the long arm of X chromosome are identified which contain putative POF candidate genes: *POF1* (Xq26-q28) (12) and *POF2* (Xq13.3-q22) (13). In POF-1, the *FMR1* gene is the most prominent candidate gene. The relationship between *FMR1* premutation status and POF disease suggests that the *FMR1* gene increases the risk of the POF (14, 15), and, based on the recent studies, the DOR pathogenesis (16, 17). Besides, the impact of shorter repeats (45-54 repeats), which are only slightly longer than normal, is less clear (17, 18). 

Epigenetics is the study of heritable changes in gene activity and expression that occur without change in DNA sequence (19, 20). Two of the most well-known epigenetic modifications are chemical modifications on cytosine residues of DNA (DNA methylation) and post-translational modification of histones associated with DNA (histone modifications) (19). Functionally, the patterns of epigenetic modifications can serve as epigenetic markers to represent the dynamic level of gene activity and expression, based on the chromatin state (21-23). These modifications play an important role in regulating gene expression by modulating the packaging of DNA in the nucleus as chromatin domains (23-25).

DNA methylation can suppress transcription through several mechanisms, including direct inhibition binding of transcription factor to gene promoters and indirect inhibition, through the induction of changes in local chromatin structure at the site of methylation. As such, methyl-CpG binding proteins (e.g., MeCP2 and MBDs) recognize methylated CpG regions, where they can acts as mediators of transcriptional repression through the association with histone deacetylases (HDACs) in repressor complexes (26-28). Histone modification is another epigenetic mechanism that is mostly known by acetylation and methylation of lysine (K) residues in N-terminal tails of histone proteins (22, 29). Histone methylation can result in the activation or the inhibition of gene expression, depending on the localization of the covalently modified lysine residue (30). For example, tri-methylation of histone 3 at lysine 4 and di/tri-methylation at lysine 9 (H3K4me and H3K9me) are particularly correlated with transcriptional activation and repression, respectively (31, 32). On the other hnad, acetylation of histones is commonly linked to active transcription (26, 27). 

Several histone modifications are reported for the *FMR1* gene. In cells with the full mutation of *FMR1*, CGG repeats are hypermethylated at H3K9 and hypomethylated at H3K4, and low levels of acetylation of histones are detected, while normally, histones H3 and H4 are hyperacetylated, H3K4 is hypermethylated, and H3K9 is hypomethylated (33-35). Previous studies have shown that the treatment of fragile X lymphoblastoid cells with the DNA methylation inhibitor 5-aza-2-deoxycytidine (5-azadC) leads to the transcriptional reactivation of the *FMR1* gene (36). In addition, the treatment of these cells with HDAC inhibitors (i.e., butyrate and tricostatin A) resulted in a modest reactivation of the *FMR1* gene. The reactivation were enhanced when the HDAC inhibitors were used synergistically with 5-azadC (37). According to our knowledge, this is the first report of the analysis and comparison of the expression levels of the *FMR1* gene in blood and granulosa cells and the evaluation of above mentioned histone modification changes of the *FMR1* gene based on the analysis of the blood cells of infertile women with DOR.

## Materials and Methods

In this study case-control study, samples for epigenetic changes and gene expression analysis were categorized into two groups: DOR patients and control groups, based on follicles number, FSH levels, and the number of CGG repeats. A total of 20 infertile women with clinically confirmed DOR conditions and the *FMR1* premutation were recruited at Department of Genetics of the Royan Institute. Any member of the DOR group had to satisfy the following conditions: Patients with 3 oocytes with a conventional stimulation protocol, *antral follicle counts* (AFC)<5-7 (2-10 mm in diameter, measured using the standardized two- dimensional technique), follicle-stimulating hormone (FSH) levels>11 IU/l at day 3 of the follicular cycle, <40 years of old, and regular menstrual cycles for the past 6 months (Table 1). Among the DOR patients, only patients with the *FMR1* gene premutation (CGG repeats >55) were enrolled as the case group. Also, 20 women with normal antral follicle numbers and serum FSH level were selected as the controls (age 37.38 ± 1.32) (Table 1). Women with abnormal karyotypes and X chromosomal mosaics were excluded from the study. All samples were collected during a one-year period (2013-2014). All patients and control subjects were Iranian, living in different places in Iran. This study was approved by the Ethics Committee for clinical research at the Royan institute and informed consent was obtained from all participants.

**Table 1:** Comparison of age, FSH level and AFC among of the patients and the controls


Group	Age	FSH level	AFC

DOR patients	31.38 ± 3.92	14.96 ± 1.83	5-6
Control	37.38 ± 1.32	<10	>7


FSH; Follicle-stimulating hormone, AFC; Antral follicle counts, and DOR; Diminished ovarian reserve.

## DNA extraction and premutation analysis

Genomic DNA was isolated from peripheral blood cells using the standard salting out method (described in (38). The 5’ UTR of the *FMR1* gene containing the CGG repeats was amplified using the polymerase chain reaction (PCR) technique by a reverse and forward primer set following Tassone et al. (39). The PCR products were separated on a 3% NuSieve 3:1 agarose gel by electrophoresis (Lonza, USA) at 33 v for 4 hours. Each DNA band were purified from the gel by High Pure PCR Product Purification Kit (Roche Applied Science, USA) and amplified by the PCR program described above. As the betaine-PCR (39) is unable to distinguish between heterozygotes of full mutation and normal homozygotes, samples that resulted in the primary PCR products with a single band were subjected to a secondary PCR screen with the R primer and the CCG-chimeric primer, instead of the F primer. Consequently, we used a chimeric CGGprimer in conjunction with betaine-PCR. The amplified product will generate a smear on the gel when there is an expanded allele present, whereas in the absence of an expanded allele no large smear will be detected. The numbers of trinucleotide repeats were confirmed by Sanger sequencing method using ABI 3730XL Capillary Sequencer. Sequencing results were compared with the sequence of a normal *FMR1* gene.

## RNA extraction and quantitative real-time polymerase chain reaction analysis

The blood and granulosa cells of 20 Iranian DOR patients (the case group) were used for RNA extraction, in order to study mRNA gene expression. Besides, patients with normal blood FSH level and more than three follicles were used as the control group (n=20). Total RNA was extracted from patient’s blood and granulosa cells using the Absolutely RNA Nanoprep kit (Aligent, USA). The integrity of total RNA was checked by denaturing formaldehyde/MOPS/1% agarose electrophoresis and then checking its purity via UV-spectrophotometry in 10 mM Na_2_HPO_4_/NaH_2_PO_4_-buffer (pH=7.0). The A260/ A280-ratio was >2.0. Two distinct ribosomal RNA bands were identified in each sample examined. To remove genomic DNA, a DNase treatment was carried out using the RNase-Free DNase Set (Qiagen, USA). We reverse transcribed RNA by QuantiTect Whole Transcriptome kit (Qiagen, USA). To exclude genomic amplification, PCR was performed with the same total RNA samples without reverse transcriptase. Products were analyzed on 4% agarose gel.

One Step Quantitative RT-PCR was performed by the 7500 Real time PCR system (Applied Bio System, USA), using Power SYBR Green PCR master mix (Applied Bio System, USA) in triplicate reaction to ensure consistency. Temperature profile of the real time-PCR consists of 95°C for 4 minutes, 40 cycles of 95°C for 10 seconds and 60°C for 30 seconds. The *FMR1* amplicon was an 89 bp product, spanning between the exons 13 and 14 of the gene. *GAPDH* was used to verify the quality of cDNA synthesis and PCR reaction (Table 2). The 2^-ΔΔCt^ was calculated for the obtained data. REST384-ß (2006) software was used to compare means between groups. 

## Chromatin immunoprecipitation coupled with real- time polymerase chain reaction 

Chromatin immunoprecipitation (ChIP) experiments were performed on the regulatory regions of *FMR1* gene [described in (38)] using Low Cell ChIP Kit (Diagenode, Belgium) and antibodies (anti histone H3 acetyl K9 antibody, anti histone H3 di-methyl K9 and anti histone H3 tri-methyl K9 (all by Abcam, UK), following the manufacturer’s instructions. Chromatin from 1×10^4^ cells was used for each immunoprecipitation reaction. Quantitative real-time PCR amplification was performed on DNA recovered from the ChIP and the total chromatin input. Five microliters of immunoprecipitated DNA (from a total 50 µl) was quantified in triplicate by real-time PCR, using Power SYBR Green PCR Master Mix (AB Applied Biosystems, USA) on a 7500 Real-Time PCR System (Applied Biosystems, USA). The primers used for PCR analysis were designed to amplify two different regions of the *FMR1* gene: the promoter region and the exon 1 near the CGG repeat. The primer pairs for ChIP experiment are listed in Table 2. Temperature profile of the real time PCR consists of 95°C 10 minutes, 40 cycles of 95°C 15 seconds and 60°C 1 minute. Data is presented as the fold enrichment of different immunoprecipitated DNA relative to a 1/100 dilution of input chromatin.

**Table 2:** Primer pairs which used in this study


	Real-time RT-PCR primers		ChIP real-time PCR primers	
Gene	Primer (5'→3')	Gene	Region	Primer (5'→3')

*GAPDH*	F: CTCATTTCCTGGTATGACAACGA	*FMR1*	Promotor	F: CGTGACGTGGTTTCAGTGTT
	R: CTTCCTCTTGTGCTCTTGCT			R: CTCCACCGGAAGTGAAACC
*FMR1*	F: GGAACAAAGGACAGCATCGC	*FMR1*	Exon 1	F: CGCTAGCAGGGCTGAAGAGA
	R: CTCTCCAAACGCAACTGGTCT			R: CTT GTAGAAAGCGCCATTGG


RT-PCR; Reverse transcription-polymerase chain reaction and ChIP; Chromatin immunoprecipitation.

## Statistical analysis of real-time polymerase chain reaction

Values were expressed as means SEM. All data were analyzed using the independent sample t test. Differences were considered statistically significant if P<0.05. 

## Results

### Premutation analysis of *FMR1* gene 

The results of CGG trinucleotide expansion in the DOR patients compared with normal individuals, has been previously reported (17). The frequency of premutation alleles was statistically higher in the DOR patients in comparison with the controls (P<0.05), but the difference in the incidence of intermediate alleles was not statistically significant between these groups.

### Expression analysis of *FMR1* gene 

**Fig.1:**
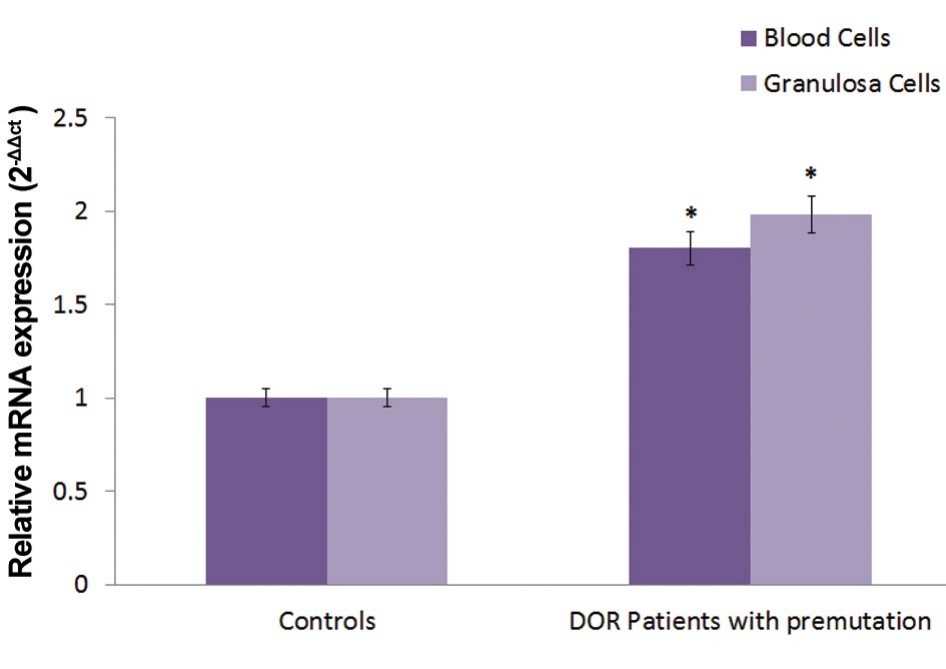
Quantitative real time polymerase chain reaction (PCR) analysis of *FMR1* mRNA levels in blood and granulose cells. The results are presented as 2^-ΔΔCt^ (mean ± SEM) relative to the *GAPDH* as the endogenous control. *; Significant difference of *FMR1* gene in the DOR patients vs. the controlgroup in P<0.05 and DOR; Diminished ovarian reserve.

Relative mRNA expression of *FMR1* gene in granulosa and blood cells of the control group and the DOR patients with *FMR1* premutation was performed using quantitative real time-PCR method. The results clearly demonstrate that the expression of *FMR1* gene in both sample types of DOR patients was about 2 fold higher than that of the control group. This increase in gene expression level was statistically significant in both types of cell samples (P<0.05, Fig .1). 

### Epigenetic profile of *FMR1* gene regulatory regions

In order to evaluate the probable epigenetic alterationsoccurred in the regulatory region of the *FMR1* gene, and the level of incorporated histone marks, we focused on known epigenetic marks of lysine 9 residue of long tailedhistone 3. Evaluated histone marks in this study were H3K9ac (an euchromatin associated mark) and H3K9me2/ me3 (heterochromatin associated marks). Data analysis in the regulatory region of *FMR1* gene demonstrated that the incorporation (presence) of H3K9ac and H3K9me2 in the promoter and the exon 1 region were significantly higher in the DOR patient in comparison with the control group (P<0.05), whereas the incorporation of H3K9me3 in the regions showed no significant difference (P>0.05, Figes.2, 3). 

**Fig.2:**
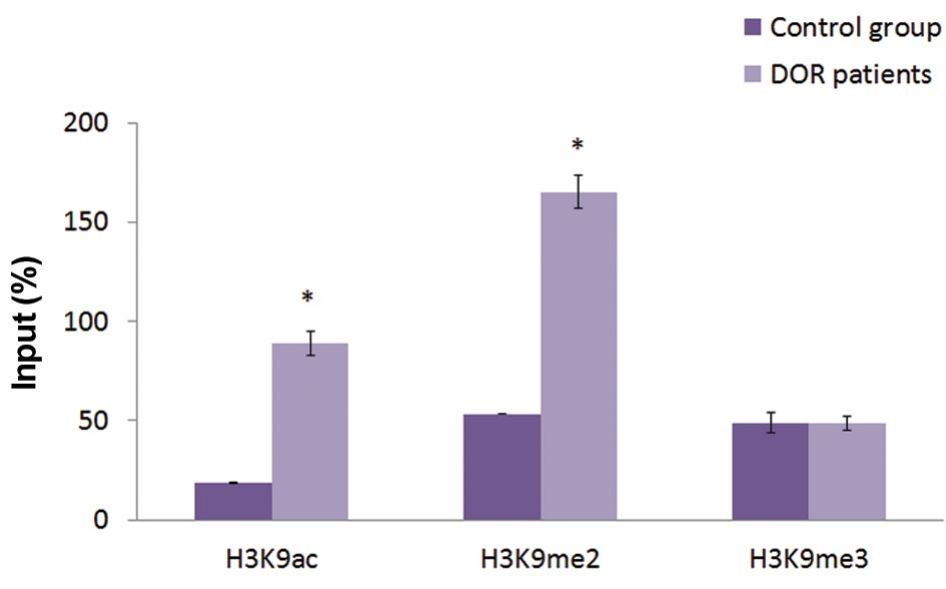
Chromation immunoprecipitation (ChIP) analysis of histone modifications in the promoter region of the *FMR1* gene in blood cells. The results are expressed relative to a 1/100 dilution of the input chromatin (mean ± SEM). *; Significant difference of incorporated histone marks in the DOR patients vs. the control group in P<0.05 and DOR; Diminished ovarian reserve.

**Fig.3:**
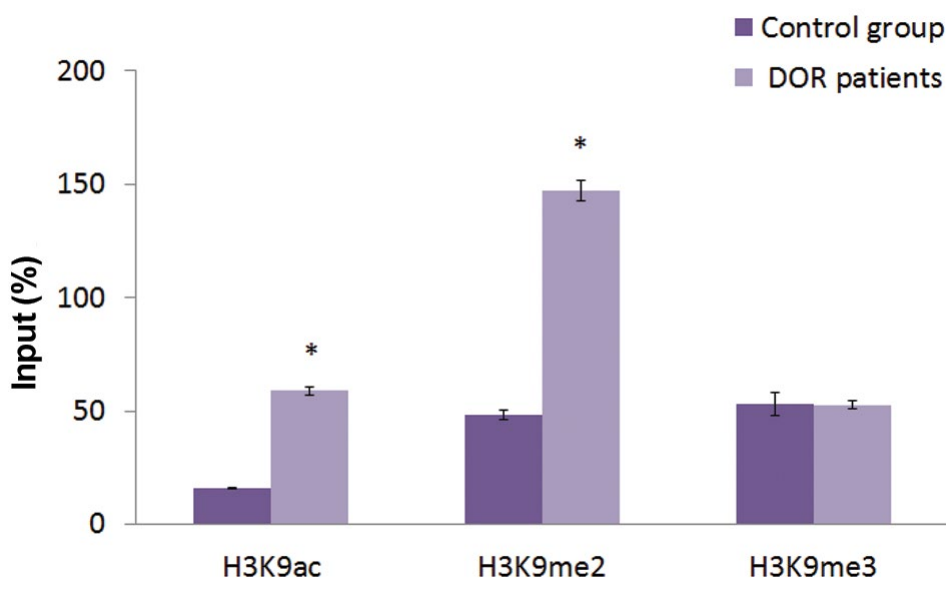
Chromation immunoprecipitation analysis (ChIP) of histone modifications in the exon1 region of the *FMR1* gene in blood cells. The results are expressed relative to a 1/100 dilution of input chromatin (mean ± SEM). *; Significant difference of incorporated histone marks in DOR patients vs. control group in P<0.05 and DOR; Diminished ovarian reserve.

### Discussion

The *FMR1* gene is transcribed in many tissues including the leukocytes. The previous studies suggested that the *FMR1* gene has a direct effect on the follicular recruitment and the ovarian reserve, implying that it has an important role in ovarian physiology and female fecundity. We investigated the epigenetic marks of methylation and acetylation of H3K9 on the regulatory region of *FMR1* gene and the resulting transcriptional activity of the gene in blood cells of patients with diminished ovarian reserve. The CGG repeat lies in the 5’-UTR of the first exon of the *FMR1* gene. Detailed analysis of the *FMR1* gene has revealed that the transcriptional regulation of the *FMR1* gene is influenced by the methylation boundary at approximately 600-800 nucleotides upstream of the CGG repeat (40, 41). The epigenetic modifications of the full mutation alleles include histone modifications, which consist of deacetylation of histones H3 and H4, low levels of lysine 4 (H3K4) methylation, and high levels of lysine 9 (H3K9) methylation. All of these changes are associated with a transcriptionally inactive heterochromatic configuration (33, 34, 42).

Several studies investigated the epigenetic modifications of the *FMR1* gene in the full mutation alleles associated with fragile X syndrome. These studies demonstrated that the transcription and the translation of a methylated full mutation can be relatively restored by treating fragile X cells with the DNA demethylating drug 5-azadC (36), whereas treatment with the inhibitors of histone deacetylases (TSA and 4-phenylbutyrate) was found to enhance the effect of 5-azadC, leading to changes in the epigenetic code of histones H3 and H4 (37, 42).

In our study, epigenetic change of the *FMR1* gene consist of H3K9ac, H3K9me2, and H3K9me3, which were examined in the promoter and the exon 1 region. Our results showed that the incorporation of H3K9ac and H3K9me2 were significantly higher in the regulatory region of *FMR1* in the DOR patient in comparison with the control groups, whereas the incorporation of H3K9me3 showed no significant difference. Based on the epigenetic profile data, it can be interpreted that although the presence of CGG repeats causes an increase in H3K9me2 level, but this hypermethylation is not a permanent state of heterochromatination. On the other hand, the dominant hyperacetylation mark observed in this region is strongly correlated with over expression of *FMR1* gene in the DOR patients rather than the control group.

### Conclusion

According to the finding obtained in this study, we propose that an increase in the number of CGG repeats to 55-200 results in the changes in the chromatin structure, which itself leads to the recruitment of histone modifier elements to this part of the genome. These epigenetic alterations cause the different expression of *FMR1* gene observed in the diminished ovarian failure.
